# Protein Kinases as Drug Development Targets for Heart Disease Therapy

**DOI:** 10.3390/ph3072111

**Published:** 2010-07-05

**Authors:** Naranjan S. Dhalla, Alison L. Müller

**Affiliations:** Institute of Cardiovascular Sciences, St. Boniface Hospital Research, and Department of Physiology, Faculty of Medicine, University of Manitoba, Winnipeg, MB R2H 2A6, Canada; E-Mail: amuller@sbrc.ca (A.L.M.)

**Keywords:** protein kinase A, protein kinase C, Ca^2+^ calmodulin protein kinase, mitogen-activated protein kinases, phosphoinositide 3-kinase

## Abstract

Protein kinases are intimately integrated in different signal transduction pathways for the regulation of cardiac function in both health and disease. Protein kinase A (PKA), Ca^2+^-calmodulin-dependent protein kinase (CaMK), protein kinase C (PKC), phosphoinositide 3-kinase (PI3K) and mitogen-activated protein kinase (MAPK) are not only involved in the control of subcellular activities for maintaining cardiac function, but also participate in the development of cardiac dysfunction in cardiac hypertrophy, diabetic cardiomyopathy, myocardial infarction, and heart failure. Although all these kinases serve as signal transducing proteins by phosphorylating different sites in cardiomyocytes, some of their effects are cardioprotective whereas others are detrimental. Such opposing effects of each signal transduction pathway seem to depend upon the duration and intensity of stimulus as well as the type of kinase isoform for each kinase. In view of the fact that most of these kinases are activated in heart disease and their inhibition has been shown to improve cardiac function, it is suggested that these kinases form excellent targets for drug development for therapy of heart disease.

## 1. Introduction

**Figure 1:**
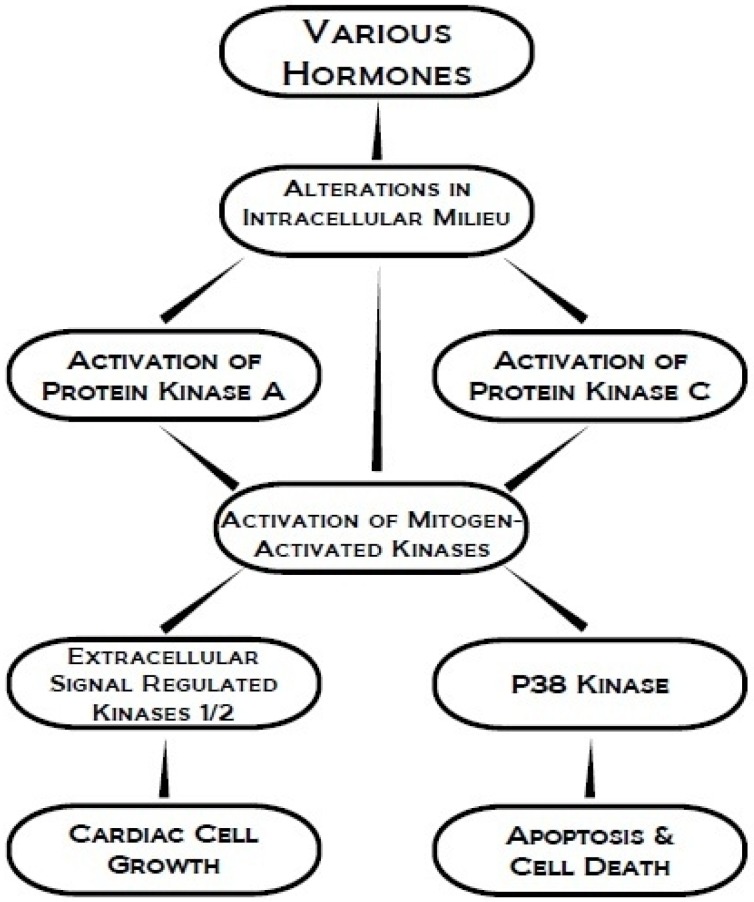
Schematic representation of the integrated involvement of some protein kinases in signal transduction for cardiac cell growth, apoptosis and cell death.

Due to their integrative role of phosphorylating specific proteins essential for modifying different biological processes, protein kinases are involved in a number of signal transduction mechanisms [1,2]. In the heart, these signal transducing proteins are engaged in the regulation of cardiac function, cation transport, myocardial metabolism, gene expression, cellular growth and cell apoptosis [3,4]. Their mode of activation involves the transfer of the γ-phosphate group from ATP to hydroxyl groups of serine/threonine residues of protein serine/threonine kinases or tyrosine residues of protein tyrosine kinases [5,6]. Protein kinases act directly by invoking a biological response immediately after activation, or by initiating branching or linear cascades of signal amplification and integration causing an eventual biological response [5]. Various hormones are known to produce a wide variety of changes in myocardial cells through the participation of different protein kinases; the integrated effects of some of these kinases are depicted in Figure 1. 

**Figure 2:**
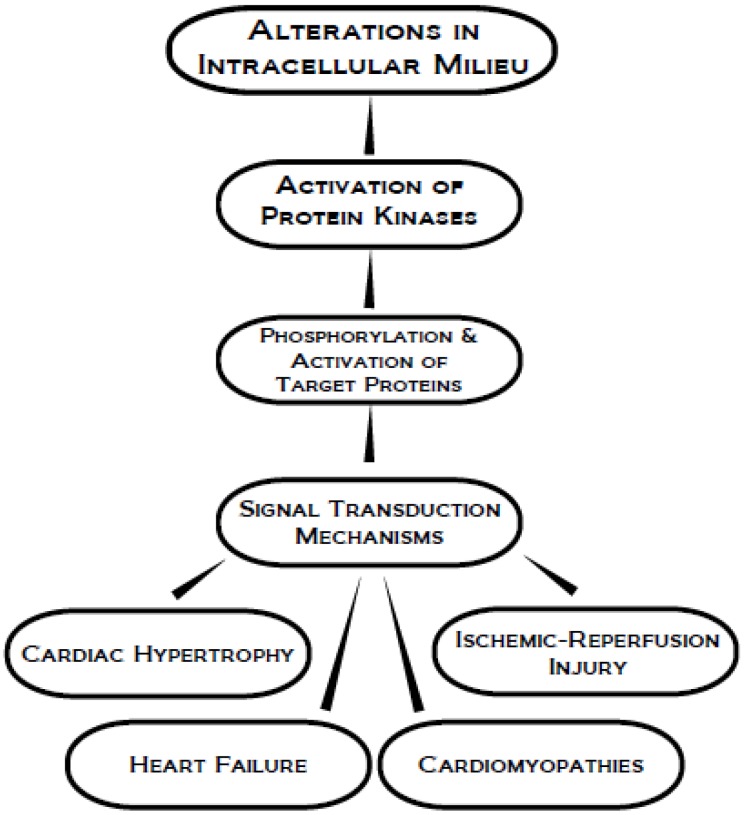
Schematic representation of protein kinase activation in signal transduction for the development of different types of heart disease.

This review is intended to focus on the currently known roles of protein kinase A (PKA), Ca^2+^-calmodulin-dependent protein kinase (CaMK), protein kinase C (PKC), phosphoinositide 3-kinase (PI3K) and mitogen-activation protein kinase (MAPK), in cardioprotection against ischemia-reperfusion (I/R) injury as well as in a number of cardiac diseases. It is also planned to discuss the participation of protein kinase-mediated signal transduction mechanisms in the development of cardiac dysfunction due to cardiac hypertrophy, heart failure and cardiomyopathies (Figure 2). Furthermore, the beneficial and detrimental effects of different inhibitors of various protein kinases will be highlighted to suggest the potential of protein kinases as targets for the future development of therapy for improving cardiac function in heart disease.

## 2. Protein Kinase A

**Figure 3:**
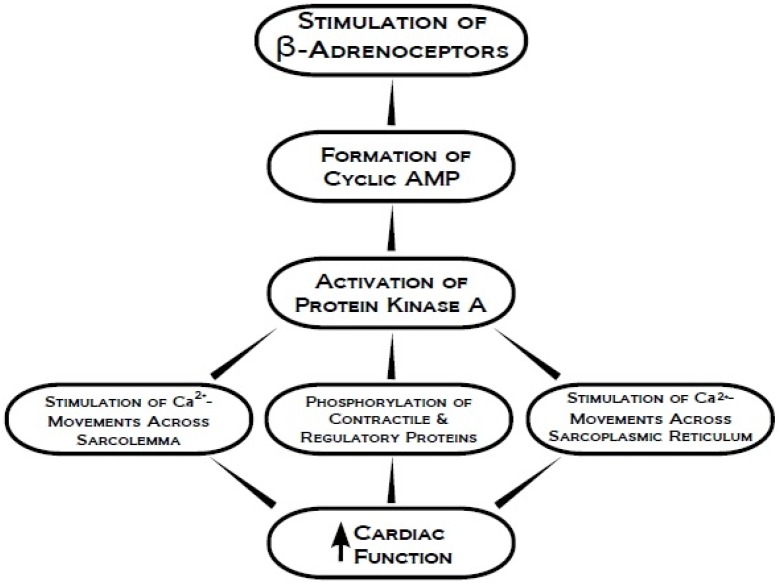
Modification of subcellular activities by the activation of protein kinase A for increasing cardiac function.

PKA is a tetrameric holoenzyme with two catalytic (C) and two regulatory (R) subunits whose activity is dependent on the concentration of cyclic AMP (cAMP) [7,8]. These regulatory subunits each bind two molecules of cAMP, which subsequently causes the R_2_C_2_ complex to dissociate and release the active C monomers [9]. PKA has been shown to phosphorylate a variety of proteins that play a significant role in regulating cardiac contractility and performance of the heart. The sarcoplasmic reticulum (SR), phospholamban (PLB), ryanodine receptor 2 (RyR2), as well as myofibrillar regulatory proteins have been extensively studied with regards to their interactions with PKA and their subsequent influence on cardiac function (Figure 3) [10,11,12,13,14]. In addition, PKA has been demonstrated to induce phosphorylation at the sarcolemmal (SL) Ca^2+^ channels and the SR Ca^2+^ pump regulatory protein PLB. It has shown to be responsive to β-adrenoceptor (β-AR) agonists, catecholamines, for stimulation in cardiomyocytes causing an elevation of intracellular Ca^2+^ concentration [15,16,17,18]. The importance of PKA in increasing cardiac contractile activity, post β-AR stimulation, is emphasized by the discovery that cardiospecific overexpression of the β_2_-AR transgene increases ventricular function *in vivo*, and modulates cardiomyocyte gene expression by phosphorylating its cAMP-response element binding protein transcription factor [19,20]. Phosphorylation of PLB by PKA has also been demonstrated to increase Ca^2+^ affinity of the SR Ca^2+ ^pump and calcium current *I*_Ca_-triggered Ca^2+^-induced Ca^2+^-release from the SR Ca^2+^ stores [21,22,23]. Since these alterations in SR PLB function were found to be independent of those in SR Ca^2+^ pump ATPase in heart failure (HF), PKA-PLB interaction is considered to be a target for drug development for the treatment of HF [24]. In addition to influencing Ca^2+^-handling by PLB, PKA has also been reported to activate RyR2. Although phosphorylation of RyR2 occurs at Ser2030 and Ser2808 [12], phosphorylation at Ser2030 is predominant upon β-AR stimulation in both normal and failing hearts, particularly concerning altered Ca^2+^ handling and cardiac arrhythmia [25]. After β-AR stimulation, SR Ca^2+^ leakage is also increased as a consequence of an elevation in PKA-dependent RyR phosphorylation [26]. It should be noted that during chronic HF, ventricular tachycardia is caused by the spontaneous release of Ca^2+^ from SR, which is partially influenced by the PKA-mediated phosphorylation of RyR2 [27]. This phosphorylation of RyR2 results in the development of intracellular Ca^2+^ overload due to excessive Ca^2+^ release from SR in this setting [27]. However, it has also been demonstrated that there is reduced phosphorylation of RyR2, as well as other PKA-dependent regulatory proteins in the failing heart [28,29] and thus some caution should be exercised while interpreting the results of RyR2 phosphorylation in HF. 

PKA plays an integral part in phosphorylating myosin and has been shown to change filament orientation and influence contractile mechanics [10]. It has been demonstrated that β-AR stimulation in both intact hearts and isolated myocardial cells can cause PKA-mediated phosphorylation of C-protein in thick myofilaments, and troponin I in thin myofilaments [30]. Specifically, it has been shown to phosphorylate cardiac myosin binding protein (cMyBP) and cardiac troponin I (cTn1) [10,11,31]. The regulation of cMyBP phosphorylation causes force generation by modulating the interaction between thick and thin filaments [10,32]; decreased level of cMyBP phosphorylation has been shown in patients with atrial fibrillation, hypertrophic cardiomyopathy and HF [33,34,35]. Phosphorylation of cTn1 decreases Ca^2+^ sensitivity of actomyosin ATPase leading to increased cross-bridge cycling and accelerated relaxation [36,37,38,39]. These observations are consistent with β-AR stimulation in the myocardium showing increased contractile force development, and augmented rate of relaxation [31]. The myofilament Ca^2+^ response has been observed to increase HF as a result in the decrease of cTn1 phosphorylation [14,40]. It is thus evident that PKA influences essential mechanisms of cardiac function and its inhibition has been shown to have dramatic effects on cardiac performance.

The inhibition of PKA has been shown to be beneficial in different studies in which PKA inhibitors, including H89 and KT5720, were observed to decrease infarct size due to myocardial infarction and prevent complications responsible for HF [41,42,43]. KT5720 inhibited the negative ionotropic and lusitropic effects and increased myocardial distensibility due to adrenomedullin (ADM) [44]. Increased ADM levels in patients with hypertension, heart failure (HF), and myocardial infarction (MI) [45,46,47,48] have been linked to increased PKA activation [49]. In HF, cytokine-induced expression of inducible nitric oxide (NO) synthetase has been suggested to contribute to myocardial depression as it has been shown to decrease cardiac myocyte contractility, sarcolemmal L-type Ca^2+^ channel activity, and sensitivity of contractile proteins to Ca^2+^ [50,51,52,53,54,55,56]. After stimulation of β-AR using isoproterenol, insulin was shown to blunt the increase in PKA activity which illustrated a potential control mechanism of PKA in maintaining Ca^2+^ homeostasis [57]. PKA inhibitor KT5720 has been reported to block the IL-1 induced NO-formation, potentially reducing the effect of damage caused by NO synthetase formation and preventing HF [42]. PKA has also been shown to enhance the phosphorylation and assembly of the proteasome *in vivo*; this was blunted by the intracoronary administration of PKA inhibitor H89 [58]. 

Reducing cell apoptosis may provide a possible therapy for preventing cardiomyocyte death because PKA inhibition decreased the extent of cell death occurring in I/R and HF [59,60,61,62,63,64]. However, inhibition of PKA has also been demonstrated to exert detrimental effects pertaining to cardiac function, specifically concerning protection from I/R injury [23,65,66,67]. In adult rats, PKA inhibitors were shown to prevent the growth hormone releasing hormone (GHRH) induced protective benefits on cardiac performance in I/R injury [65]. In another study, the cardioprotective effect on nitric oxide-induced cytotoxicity in H9c2 cardiac cells was prevented following treatment with PKA inhibitors, H89 and KT5720 [68]. There has also been evidence suggesting that NO-induced apoptosis of cardiomyocytes is protected by roflumilast, a PDE4 inhibitor, via the activation of the PKA and Epac dual pathway [66]. It is pointed out that NO is known to affect cardiomyocytes by stimulating guanylyl cyclase and formation of cyclic GMP, which, like cAMP, activates protein kinase but produces effects opposite to those by cAMP. Furthermore, PKA activation was found to modulate the activity of cytochrome C oxidase during experimentally-induced ischemia and demonstrated that the PKA inhibitor, H89, reversed the phosphorylation and kinetic properties of cytochrome C oxidase and thus protected the heart against ischemic heart injury in rabbits [69]. 

## 3. Ca^2+^-Calmodulin Dependent Kinase

**Figure 4:**
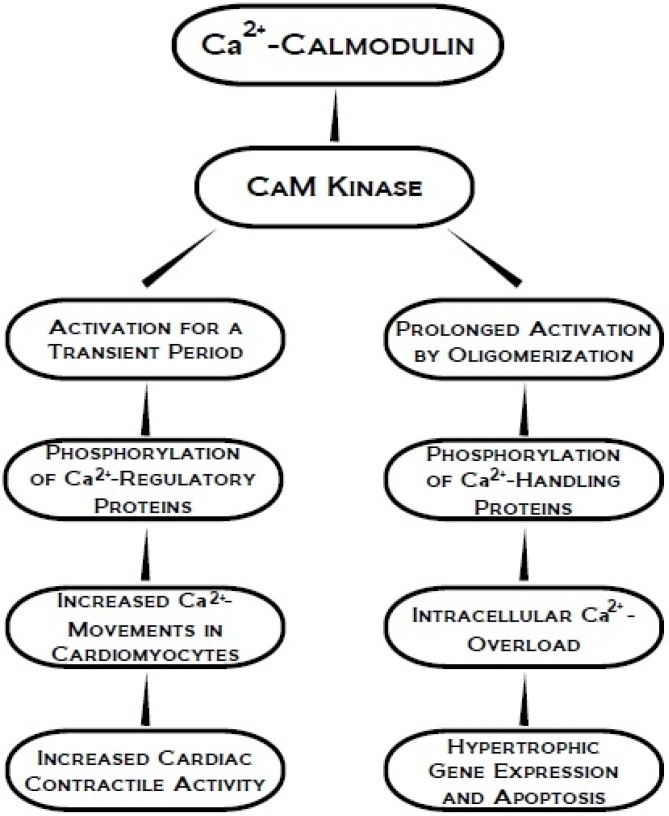
Transient and prolonged activation of CaM kinase in phosphorylating Ca^2+^ cycling proteins for the occurrence of beneficial and detrimental effects on the heart.

CaMK is another protein kinase which is activated by calcium and calmodulin, a Ca^2+^-binding protein. CaMKII is a highly conserved Ser/Thr protein kinase encoded by four genes α, β, γ, and δ [70,71]. Although this signal transducing protein kinase is activated by Ca^2+^-calmodulin for a transient period, the resulting oligomerization of its subunits is associated with a prolonged period of activation [70,72,73]. It has been reported that α, β, and δ subunits of CaMK II are hetero-oligomerized in variable domain spacers that alter the relative position of catalytic domains and influence both the rate of inter-subunit autophosphorylation and Ca^2+^ sensitivity [74]. CaMKII has also been observed to phosphorylate SR Ca^2+^ release channels or RyRs, PLB, and SL L-type Ca^2+^ channels [75,76,77] and is thus believed to modulate excitation-contraction coupling by phosphorylating these Ca^2+^ regulatory proteins subsequently influencing cardiac function [77,78]. However, a key factor to consider is that Ca^2+^ is initially necessary to activate CaMKII, but Ca^2+^ is not required for prolonged activation which may have a potentially detrimental effect on cardiac function. Prolonged activation of Ca^2+^ handling proteins by CaMKII due to β-AR stimulation has been reported to induce hypertrophic gene expression and apoptosis [71,72,79,80,81,82]. Signal transducing events associated with both beneficial and detrimental effects involving CaMK activation for transient and prolonged periods are represented in Figure 4. It has also been shown that the angiotensin II-induced apoptotic cascade may be controlled by reactive oxygen species (ROS)-dependent CaMKII activation, particularly at sub-diastolic Ca^2+^concentrations which imply that CaMKII may become activated at very low Ca^2+^ levels when exposed to ROS [83]. Taken together, it is evident that the beneficial or detrimental effects of the activation of CaMKII-mediated pathway are dependent upon the intensity and duration of stimulus.

As with PKA, there have been numerous studies showing both the beneficial and detrimental effects of inhibiting CaMKII in cardiac disease. CaMKII activation may trigger lethal ventricular arrhythmias with increased oxidative stress [84,85]. Inhibition of CaMKII has been shown to suppress isoproterenol-induced arrhythmias *in vivo* [86,87], as well as prevent the increased effects of stress-induced β-AR stimulation on cardiac contractility and Ca^2+^-handling [88]. CaMKII has been linked to atrial fibrillation as a result of its observed increased phosphorylation of RyR2 that subsequently causes Ca^2+^ leakage [89]. It has also been suggested that pressure overload can induce CaMKII-mediated activation of protein synthesis resulting in the development of cardiac hypertrophy [90], which is adaptive in nature during the initial period but then becomes pathophysiologic over a prolonged period. Specifically, the δ isoform of CaMKII has been shown, *in vivo*, to be involved with pathological cardiac remodeling in a pressure-overload mouse model [91]. It is therefore important to consider the roles of different isoforms of CaMKII as they may mediate different aspects of cardiac function. After aortic constriction-induced cardiac hypertrophy, CaMKII δ and γ levels are increased, although the highest degree of autophosphorylation occurs in CaMKII δ [92], implying that inhibition of the δ isoform would have a greater effect in reducing cardiac hypertrophy without causing extensive disruption to normal CaMKII function. A study by Peng *et al*. [93] demonstrated a functional difference between CaMKII δB and δC, showing that δB isoform suppresses cardiomyocyte apoptosis triggered by multiple death-inducing stimuli whereas δC isoform stimulated a reverse effect. It should be noted that the δB isoform contains a nuclear localization signal whereas the δC isoform remains in the cytosol [94]. Overexpression of CaMKII δB was shown to protect cardiomyocytes against oxidative stress, hypoxia, and angiotensin-II induced apoptosis, potentially through inhibition of δC [94]. Acute CaMKII δC overexpression in mice, observed in cardiac hypertrophy and reduced systolic function [86], has also been shown to alter RyR function, resulting in enhanced SR Ca^2+^ leakage. The enhancement of RyR Ca^2+^ release could possibly lead to increased chances of ventricular arrhythmia [86]. This detrimental effect was blocked by CaMKII inhibitors KN93 and autocamtide-2-related inhibitory peptides [95]. These observations support the view that the beneficial or detrimental effects of CaMKII signal transduction pathway may depend upon the type of isoform of the enzyme involved in this process. 

CaMKII δ3 has also been shown to induce cardiomyocyte hypertrophy via the apoptosis signal-regulating kinase (ASK1) in mammalian cells, where KN93 prevented Ca^2+^-induced ASK-1 phosphorylation [96]. These data suggest that the inhibition of CaMKII, particularly isoform δ, could result in the prevention of cardiac hypertrophy. Despite the majority of literature indicating that CaMKII is the most involved CaMK in cardiac hypertrophy, CaMI and CaMKIV have also been shown to induce hypertrophic responses in cardiomyocytes *in vivo* [97,98]. Overexpression of CaMKIV in mice resulted in increased left ventricular end-diastolic diameter and decreased fractional shortening [97]. On the other hand, inhibition of CaMK may not be so beneficial in other forms of cardiac diseases. For example, it has been shown that KN93 inhibits ischemic preconditioning-mediated improvement in recovery of cardiac function after I/R, illustrating the requirement for CaMKII activation during preconditioning [99,100]. Inhibition of CaMK in the failing heart may not be beneficial, as alterations in SR CaMK-mediated phosphorylation have been shown to be partially responsible for the impaired function of SR in the heart due to an observed decrease in SR protein contents and SR CaMKII activity [101]. Despite the evidence indicating involvement of CaMKII δC in the development of cardiac hypertrophy, it has been shown to phosphorylate calcineurin and inhibit its activity. It is pointed out that calcineurin is known to cause cardiac hypertrophy, mechanical dysfunction, arrhythmias and premature death [102] suggesting that its inhibition by active CaMKII δ may be more effective than inhibition of the inactive form of CaMKII δ, per se. 

## 4. Protein Kinase C

PKC is a Ca^2+^-activated, phospholipid-dependent enzyme that consists of 12 distinct isoforms, each containing a highly conserved carboxyl terminal kinase domain that includes an ATP-binding site [103]. In the heart, the majority of PKC appears in the α and β forms [103,104], with the most dominant form expressed in the human heart being isoform α [105]. Inactive PKC isoforms present in the cytosol translocate to membranes in a Ca^2+^-dependent active form [106]. There are three distinct classes of PKC, conventional PKC (cPKC), novel PKC (nPKC), and atypical PKC (aPKC). The difference between these families is based on their mode of activation. cPKCs are activated by both diacylglycerol (DAG) and Ca^2+^ and consist of the α, βI, βII, and γ isoforms. nPKCs are activated by DAG independently of Ca^2+^ and consist of the δ, ε, η and θ isoforms. aPKCs are activated by phosphoinositide-dependent kinase-1, independently of Ca^2+^, and consist of the ζ and λ isoforms [107,108,109]. PKC has significant involvement in a variety of signaling pathways mediating heart function so it is important to consider the type of cardiac dysfunction/disease along with the isoform involved before ruminating inhibition, as its influence can be either beneficial or detrimental to cardiovascular activity. PKC has been shown to have increased myocardial activity associated with cardiac dysfunction in the diabetic heart [110]. The PKC isozymes α, β, ε, and ζ, are increased in both homogenate and cytosolic fractions of the diabetic heart, as well as increase Ca^2+^-independent activity, illustrating association with subcellular alterations and cardiac dysfunction in the diabetic heart [109]. 

**Figure 5:**
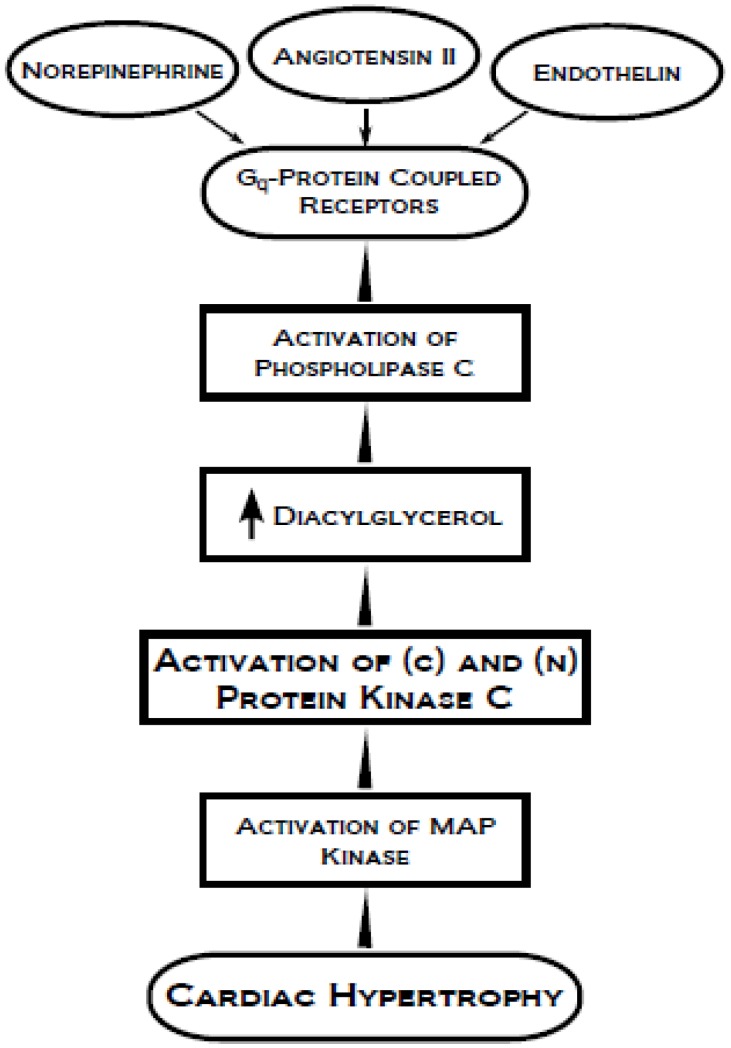
Signal transduction mechanisms involving the activation of conventional (c) and novel (n) protein kinase C and MAP kinase for the development of cardiac hypertrophy.

**Figure 6:**
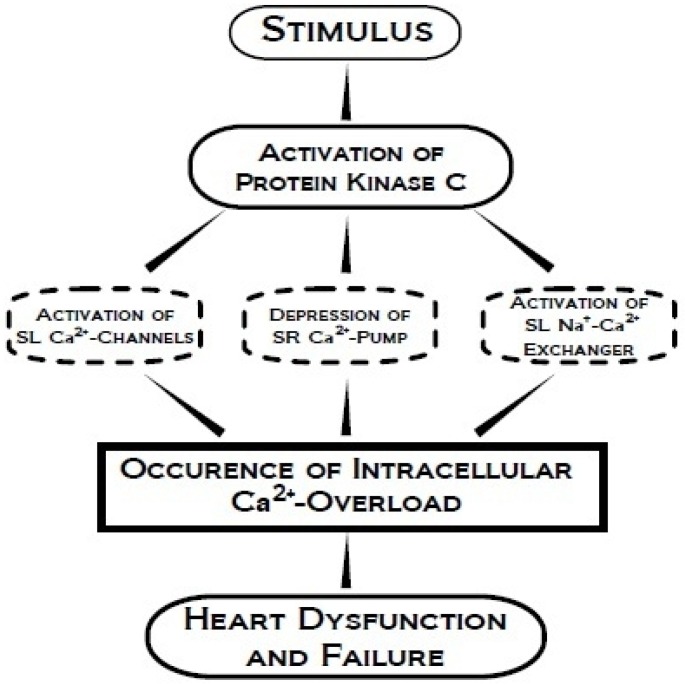
Schematic representation of the signal transduction pathway involving the activation of protein kinase C, occurrence of intracellular Ca^2+^ overload and cardiac dysfunction.

Various hormones are known to induce cardiac hypertrophy through the involvement of PKC activation and associated signal transduction mechanisms (Figure 5). Isozyme-specific PKC activity has been shown to lead to ventricular hypertrophy. Increases in PKC activity and concentration, particularly of isozymes PKC-β_1,2_ and PKC-ε, during the development of left ventricular hypertrophy induced by pressure overload occurs in membrane and nuclear-cytoskeletal fractions [111]. Another study showed an increase in PKC-δ isozyme, but not of PKC-α or PKC-ε, in volume-overload-induced left ventricular hypertrophy; however, PKC-α was found, along with PKC-δ, to have increased enzyme activity and protein expression in right ventricular hypertrophy [112]. PKC-ε has also been shown to be responsive to mechanical deformation in adult guinea pig myocardium, as the stretch caused by cardiac hypertrophy was indicated to lead to increased protein synthesis in cardiac muscle which subsequently activated the phospholipase C pathway, due to angiotensin II [5,113,114,115,116]. An additional study also found that PKC-δ autophosphorylation was increased prior to left ventricular hypertrophy development as well as during the transition to HF. PKC-α expression was found to be increased during the transition to HF, but remained unaffected during the induction of cardiac hypertrophy [117]. The signal transduction mechanisms involving the activation of PKC, occurrence of intracellular Ca^2+^ overload and development of cardiac dysfunction and HF are depicted in Figure 6. Thus, it appears that the beneficial or detrimental effects of changes in PKC signal transduction may be associated with the type of PKC isoform.

PKC inhibition has been shown to be beneficial in a number of ways, including preventing diabetes-induced abnormal cardiomyocyte mechanics [118] and enhancing cardiac contractility [105]. PKC-α and PKC-β isoforms in diabetic hearts showed increase expression which was associated with loss of contractility and subsequent HF due to their regulatory effect on cardiac contractility and Ca^2+^-handling in cardiomyocytes [105,119,120,121,122]. Treatment with breviscapine decreased the activity of these PKC isoforms, and subsequently inhibited cardiomyocyte apoptosis induced via I/R injury and was shown to improve cardiac structure and function with satisfactory tolerance and safety [119,123,124]. PKC-β inhibition by ruboxistaurin has also been shown to attenuate cardiac diastolic dysfunction, myocyte hypertrophy, and preserve cardiac contractility in diabetic cardiomyopathy [125]. Aldosterone was reported to induce vasoconstriction via PKC-dependent pathways that ultimately decreased cardiac contractility and metabolic functions in the ischemic heart. An inhibitor of PKC, GF109203X, attenuated the effect of aldosterone on impaired cardiac function [126]. Ruboxistaurin, a PKC inhibitor, was also found to attenuate pathological fibrosis and impaired cardiac function after induced MI [127]. Our laboratory showed that increased PKC activity in cardiac dysfunction due to MI was associated with increased expression of the PKC-α, -β and -ε isoforms [128]. In wild-type mice undergoing 10 weeks of pressure-overload, this PKC inhibitor reduced ventricular dilation, enhanced ventricular performance, and reduced fibrosis [129]. Furthermore, in a gene knock-out study, Liu *et al.* [129] demonstrated that PKC-α -/- mice undergoing long-term pressure overload exhibited increased Ca^2+^ transients and SR Ca^2+^ load and were less susceptible to HF, whereas the PKC-β/γ -/- mice displayed severe HF. When treated with ruboxistaurin, there was increased contractility in PKC-β/γ -/- mice, but not PKC-α -/- mice [129]; these studies highlighted the importance of PKC isoform-specific inhibitors. This point is further emphasized by a study showing that the targeted inhibition of PKC-α using Ro-320432 enhanced cardiac contractility in two different models of HF *in vivo*. In addition, it was demonstrated that PKC-α-specific inhibitor Ro-318110 augmented cardiac contractility and restored pump function in the lim-/- mouse model of HF [105]. Gö 6983, an inhibitor of different PKC-isoforms, was shown to prevent intracellular Ca^2+^ accumulation when administered at the beginning of reperfusion and attenuated the post-I/R cardiac contractile dysfunction [130,131]. When investigating polymorphonuclear leukocytes (PMN) vascular adherence and infiltration into post-reperfused cardiac tissue, a PKC-βII inhibitor was found to attenuate the PMN-induced post-I/R cardiac contractile dysfunction by inhibiting PMN-superoxide release and increasing endothelial NO release [132]. PKC-ζ inhibition has also been found to increase endothelial NO release and inhibit superoxide release during PMN-induced post-I/R while attenuating their infiltration into I/R myocardium [133]. 

Not only must one consider the variety of isoforms of PKC when targeting it for inhibition, but a study by Hahn *et al.* [134] has shown that the degree of inhibition is also important. Their investigation studied how various levels of PKC-δ V1 (δ-specific PKC translocation inhibitor peptide fragment) expression influences cardiac function. They ascertained that low levels of PKC-δ V1 resisted post-ischemic dysfunction with no measureable variation on basal cardiac structure, function or gene expression. However, the increase of PKC-δ V1 expression led to decreased ischemic protection, abnormal gene expression, and cardiomyocyte deformities, to the point of depressed cardiac contractile function and the formation of cardiomyocyte protein aggregates [134]. Although there is an extensive amount of work which has been carried out to suggest the potential benefits of PKC inhibition, it is still important to consider the negative influences of inhibiting PKC on cardiac function. PKC inhibition by chelerythine has been demonstrated to prevent the preconditioning of myocardial function and attenuate the diminution of lipid peroxidation due to ischemic-preconditioning [135]. Additionally, PKC activation has been suggested to be crucial in the mechanism of protection against contractile dysfunction by preserving diastolic function and the ratio of metabolic: mechanical efficiency to contractile function [136]. Although beneficial effects of inhibiting PKC activation by angiotensin II have been illustrated, it has been shown that angiotensin II mediated activation of PKC can also limit infarct size in rabbit hearts due to MI [137]. Inhibition of PKC-ε in mice has been shown to increase cardiomyocyte size and impair left ventricular functional shortening, as well as cause lethal dilated cardiomyopathy [138]. In the rabbit heart, PKC-ε has been observed to be involved in ischemic pre-conditioning [139]. Thus a balance between negative and positive effects of PKC inhibition must be taken into account while considering this site as a target for therapeutic venture. 

By far the most significant effect of PKC activation, particularly PKC-ε, is its involvement with cardioprotection from I/R injury [140]. Activation of PKC-ε by a peptide has been shown to have cardioprotective effects by enhancing vascular endothelial NO release when given prior to inducing ischemia [141], and when administered during preconditioning, this peptide improved cardioprotection against I/R injury [142]. The cardioprotective effects of opioid receptor stimulation may potentially be influenced by PKC-ε translocation from the cytosol to the membrane as reduced cell injury/death from lethal ischemia was observed after translocation of the enzyme [143]. PKC-ε has also been reported to prevent hyperglycemia-induced apoptosis whereas other cardiac PKC isozymes modulate hyperglycemia-induced apoptosis [144]. PKC-ε has been observed to exert cardioprotection in diabetic ψε-RACK (ε-agonist) mice via inhibition of the hyperglycemia apoptosis signal, attenuation of hyperglycemia-mediated oxidative stress, and preservation of parameters of the left ventricular pump function. The study additionally unveiled PKC-ε undergoing selective trafficking to membrane and mitochondrial compartments, as well as inactivating Bad protein and inhibiting cytochrome *c* release for preventing cell death [145]. Fibroblast growth factor-2 exhibited cardioprotective effects via the PKC-dependent pathway as illustrated by decreased translocation of PKC-δ reducing post-ischemic contractile dysfunction and MI [146]. PKC-α and -δ isoforms may also play a role in sildenafil-induced cardioprotection against I/R injury in the rabbit heart because their translocation from the cytosol to the membrane was affected [147]. Although it is commonly held that inhibition of PKC-ε would prevent cardioprotection, activation of the PKC-δ isoform showed increased damage by ischemia both *in vitro* and *in vivo*, emphasizing the need for selective PKC inhibitors [148]. As discussed previously regarding PKA and CaMKII, the selectivity of PKC isoforms is significant in considering therapeutic options for I/R injury when evaluating whether inhibition is favourable or unfavourable for cardioprotection. 

## 5. Phosphoinositide 3-Kinase

PI3K is a heterodimeric complex of p85 and p110 proteins that contain two SH2 domains, thought to function as adaptor motifs linking tyrosine kinases to their substrates [149,150,151,152]. The p110 catalytic and p85 regulatory subunits form obligate heterodimers *in vivo* [153]. The catalytic domain of PI3K has a bilobal organization with an ATP-binding pocket in a cleft between N- and C-lobes, and the catalytic residues in the phosphate binding subsite [154]. All PI3K inhibitor complexes reported are type I inhibitors, which occupy the adenine region making hydrogen bonds to the hinge region of PI3K, including the pan-specific LY294002 and PI3Kγ-specific AS-604850 [155,156]. Different isoforms of PI3K have also been shown to mediate different aspects of cardiac cell functions. For example, PI3Kα appears to play a role in mediating changes in cell size whereas PI3Kγ has been shown to inhibit cAMP production and negatively regulate cardiac contractility [157,158]. To further emphasize the dichotomy between PI3K inhibition being either beneficial or detrimental for cardiac function, there is a need to note the type of cardiac dysfunction when considering inhibitory measures as therapy. 

Inhibition of PI3K was demonstrated to be beneficial in a variety of cardiovascular diseases. It has been shown to reduce ischemia-induced arrhythmias [159] and inflammation after MI [160]. In an experiment dealing with chronic pressure overload and PI3K overexpression, it was found that inactive PI3K displaced the endogenously active PI3K and subsequently caused a delay in the development of cardiac dysfunction [161]. Membrane PI3K activity has been shown to contribute to HF [162,163]. In failing pig hearts, PI3K activity associated with βARK1, was increased; however, after adenoviral gene transfer of the PI3K domain (the phosphoinositide kinase domain that directly associates with βARK1), there was a decrease in the receptor-localized PI3K activity which resulted in nearly normal cardiomyocyte contractility [163]. In pressure overload-induced HF, preventing β-AR association with PI3K resulted in alleviating the development of HF [164]. Preventing this association via PI3K inhibition may provide preservation of β-AR signaling resulting in preventing complete HF as a consequence of cardiac hypertrophy. Tumour necrosis factor (TNF) has been shown to activate PI3Kγ leading to events that subsequently cause decreased cardiac function, associated with superoxide radical production. The activation of PI3Kγ leads to adverse myocardial remodeling and possible inhibition of this activation could prevent decreased cardiac performance in the failing heart [165]. The PI3Kγ isoform has been reported to have increased expression under pathological pressure overload [166] suggesting that its inhibition may prevent subsequent cardiac hypertrophy. In an experiment dealing with exercise-induced cardiac hypertrophy, it was found that a deletion of the p85 regulatory subunit of PI3K not only notably attenuated Akt signaling, but resulted in reduced heart size and altered cardiac gene expression while still exhibiting normal contractility and myocardial structure [167]. These observations indicate the necessity of considering both isoform- and subunit-specific inhibition in various cardiovascular malignancies. 

Inhibition of PI3K could also be detrimental to cardiac function. After ischemic reperfusion in isolated rat hearts, upregulation of G protein-coupled estrogen receptor by G-1 was observed to improve the cardiac function recovery and reduce infarct size, where beneficial effects were abolished upon treatment with PI3K inhibitor, wortmannin [168]. Wortmannin has also been shown to prevent the functional recovery in epoxyeicosatrienoic acid treated hearts [169]. In myocardial I/R injury, the anti-apoptotic effect appears to be promoted by the PI3K/Akt pathway after adrenomedullin administration by accelerating the phosphorylation of Akt; this action was prevented upon treatment with wortmannin [170]. 

An increase in PI3K activity has also been demonstrated to be effective in reducing atrial fibrosis and improving cardiac conduction in dilated cardiomyopathy transgenic mice. The reduction in PI3K activity, specifically the regulatory subunit β 110 α, was found to increase the susceptibility to atrial fibrillation in patients [171]. It is also important to consider the differences between various regulatory units as it has been shown that PI3K p110 α, unlike PI3K p110 β, regulates voltage-dependent L-type Ca^2+^ channels in cardiac myocytes localized on the cell surface. Myocytes were found to have contractile defects *in vivo* when lacking PI3K subunit p110 α [172]. Increased PI3K p110 α activity has been shown to delay the progression of heart disease by attenuating pathological growth and preventing fibrosis in a pressure-overload model mouse of HF [173]. Another study showed that PI3K p110 α may induce physiological growth in response to exercise, but is not responsible for pathological growth due to pressure overload [174] suggesting that its inhibition may not be effective in preventing hypertrophy but could be detrimental to athletic individuals by preventing their heart from being able to compensate for an increased workload. Activation by the growth factor midkine has been shown to prevent cardiac remodeling post MI via the PI3K/Akt pathway; this event has been suggested to be an effective enhancement of angiogenesis [175]. The progression of dilated cardiomyopathy was observed to be prevented by the PI3K/Akt pathway after administration of darbepoetina α; an agent known to improve cardiac function [176]. Complete absence of PI3K-γ causes MI whereas the complete absence of PI3K-β showed a severe decrease in contractile performance in mice [177] indicating the differential role of PI3K isoforms in inducing different effects.

The PI3K/Akt pathway is another protein kinase signal transduction mechanism which is involved in cardioprotection. Heat shock protein 90, shown to be protective against cellular stress prevented apoptosis partially mediated by the PI3K/Akt pathway in cardiomyocytes subjected to hypoxia [178]. Additionally, estrogen receptor β has been shown to provide myocardial protection by increasing PI3K/Akt activation as indicated by decreased expression of caspase 3 and caspase 8 and sustained Bcl-2 levels [179]. In diabetic rat myocardium, peroxisome proliferator-activated receptor α has been shown to provide protection against I/R injury by activating the PI3K/Akt pathway, as cardioprotection was attenuated by treatment with PI3K inhibitor, wortmannin [180]. In diabetic myocardium, it has also been reported that the ischemic preconditioning stimulus has to reach a crucial level of Akt activation in order to allow for the occurrence of cardioprotection. A minimum of three cycles of IPC must occur after activation of the insulin receptors in order to achieve significant levels of cardioprotection [181,182] indicating the importance of stimulus intensity in determining the response. The PI3K/Akt survival pathway has also been shown to be activated by pretreatment with erythropoietin against doxorubicin-impaired heart function and cardiomyocyte apoptosis and oxidative stress [183]. In addition, preconditioning using hydrogen sulphide appears to provide cardioprotection by activating the PI3K/Akt pathway [184]. In rats adapted to chronic intermittent hypobaric hypoxia, it was shown that inhibition of the PI3K/Akt pathway was able to attenuate ischemia-induced arrhythmias however, it exacerbated myocardial injury [159]. 

Another study showed that chronic activation of Akt could potentiate myocardial complications, however restoration of PI3K after I/R injury rescued left ventricular function and decreased the extent of injury. This illustrates the possibility that PI3K-dependent and Akt-independent effects are required for more complete cardioprotection [185]. In both early reperfusion and prolonged ischemia, isoflurane has been shown to involve the PI3K pathway for salvaging myocardium from infarction [186]. In addition, the ischemic post-conditioning protection also appears to activate PI3K-Akt [187]. Tang *et al*. [188] found that the PI3K/Akt pathway appears to be involved in the migration of vascular endothelial growth factor (VEGF)-mediated cardiac stem cells. An increase in PI3K/Akt activity was noted along with an increase in the number of cardiac stem cells occurring in infracted regions resulting in an improvement in cardiac function. 

Thus, it is evident that the PI3K/Akt pathway is intimately involved in cardioprotection and the use of its inhibitors may produce detrimental effects on cardiac function. It should be noted that the Akt isoforms 1 and 2 are targets of PI3K and demonstrate similar substrate specificities, however Akt1 has been shown to be essential for the physiological growth of the heart whereas Akt2 is essential for maintaining normal glucose metabolism [189,190,191]. Interestingly, it was observed that Akt1 can contribute detrimentally to cardiac hypertrophy whereas, in contrast, Akt2 slightly attenuated ischemia-induced cardiomyocyte apoptosis [190,191].

## 6. P38 Mitogen-Activated Kinase

MAPK is a family of proline-direct serine/threonine specific protein kinases. There are four distinct families of kinases: extracellular signal regulated kinases (ERK1 and 2), c-jun *N*-terminal kinases (JNK1-3), p38 MAPK, and ERK5. They share an overall 60–70% similarity with variations present between their activation loop sequences and sizes [192]. Activation of MAPK upon Ca^2+^ depletion and Ca^2+^ repletion has been associated with cardiac dysfunction and apoptosis; these changes were attenuated by pretreatment with taurine [193]. The activation of p38 MAPK α-isoform has been shown to facilitate apoptosis but overexpression of the p38 MAPK β isoform was found to contribute to the development of cardiac hypertrophy [194]. The inhibition of MAPK, in particular p38 MAPK, has been demonstrated to exert beneficial effects on cardiovascular health, including involvement in protection against myocardial ischemia damage [195,196,197,198,199,200,201,202]. A p38-MAPK knockout has shown to be resistant to I/R injury [203]. In spontaneously hypertensive stroke-prone rats fed a salt-fat diet, treatment with a selective p38 MAPK inhibitor improved survival and attenuated dyslipidemia, hypertension, and cardiac remodelling [204]. Selective MAPK inhibitor, SB203580, was observed to attenuate amyloidogenic Ig light chain-induced oxidative stress, as well as cellular dysfunction and apoptosis. This study reported that transforming growth factor β-activated protein kinase-1 binding protein-1 (TAB1)-mediated p38 MAPK-α isoform autophosphorylation may be involved in the onset of cardiac dysfunction and subsequent HF [205]. Treatment with MAPK inhibitor, SB203580, has been shown to attenuate myocardial fibrosis and LV remodelling in a myocardial ischemia rat model [206]. It has also been found that p38 MAPK may contribute to cardiac injury during I/R, particularly in patients with type 2 diabetes [207]. However, it was observed that inhibition of p38 MAPK with SB 202190 did not alter the functional parameters of diabetic cardiomyopathy induced by glucose, in spite of an increase in p38 MAPK phosphorylation [208]. This may indicate an involvement of p38 MAPK in cardiac dysfunction, and perhaps a different MAPK inhibitor is required for the attenuation of functional changes in diabetic cardiomyopathy. MAPK signaling has been shown to be involved in vascular smooth muscle cell hypertrophy and proliferation and it has led to the suggestion of inhibiting both p38 MAPK and p42/44 MAPK to reverse vascular dysfunction in HF patients [209]. An increase in ROS has been linked to increased MAPK activation and thus causing subsequent myocyte damage [210]. In a study investigating the role of changes in ERK1/2, p38α MAPK, and JNK1/2 activities, it was found that the activation of ERK1/2 promoted cardiomyocyte survival after pressure overload, however, p38β MAPK and JNK1/2 were found to promote pathological cardiac remodeling [211]. A highly selective and orally administered p38 MAPK inhibitor, SB239063AN, has been demonstrated to reduce superoxide generation, blunt hypertension, and cardiac hypertrophy in Sprague-Dawley rats treated with angiotensin II [212]. 

p38 MAPK has been shown to be involved with the expression of pro-inflammatory cytokines and inflammatory responses responsible for cardiac dysfunction development [213]. Inhibition of p38 MAPK using FR167653 was found to prevent neutraphil accumulation in the heart, inflammation-related injuries and frequent ventricular arrhythmia. In diabetic cardiomyopathy, cytokine levels and fibrosis are increased, and it was found that inhibition with p38 MAPK inhibitor, SB203580, restored cytokine levels to normal, prevented inflammation, and improved systolic function [214]. Combination treatment with SB203580 and FR167653 in BIO 14.6 hamsters with cardiomyopathy was shown to attenuate myocardial fibrosis and left ventricle chamber dilation, as well as reduce the heart to body weight ratio [215]. In a rat model where myocardial damage was induced by L-NAME/ANG II/NaCl, inhibition of MAPK using SD282 was observed to increase stroke volume, cardiac output, ejection fraction, and stroke work showing a significant improvement in cardiac function [216]. In endotoxin induced myocardial contractile dysfunction, p38 MAPK inhibition was found to decrease myocardial TNFα, IL-1α, IL-1β, and IL-6 production, reduce inflammation and improve cardiac function [217,218]. Myocardial functional impairment induced during TNFα release was limited by treatment with p38 MAPK inhibitor, SB203580 [219]. This inhibitor was also shown to be effective in decreasing myocardial apoptosis after I/R injury [200]. 

In view of both detrimental and beneficial effects of different types of MAPK, a great deal of caution should be exercised while using various inhibitors for these targets. Activation of ERK1/2 after pressure overload has been shown to promote cardiomyocyte survival [211]. Since over-expression of Hsp27 has cardioprotective effects in I/R injury, inhibition of p38 MAPK attenuated the insulin-induced phosphorylation of Hsp27 and prevented its cardioprotective role in reducing infarct size [220]. Pre-treatment with adenosine A_1_/A_2A_ (AMP579) has been shown to reduce myocardial stunning by activation of the p38 MAPK pathway [221,222] whereas inhibition by SB203580 prevented the attenuation of myocardial stunning [221]. When p38 MAPK is knocked-out in mice, they develop heart dilation and cardiac dysfunction earlier when compared to control undergoing pressure overload treatment [215]. A constitutively active form of MAPK-kinase 5 has been reported to accelerate the recovery of left ventricular developed pressure after I/R by increasing BMK1 activation and causing hyperphosphorylation of connexin 43 [223]. p38 MAPK activity has also been inversely correlated with stretch-induced HF where the decrease in p38 MAPK activity led to an increase in left ventricle diameter [224]. Direct inhibition of MAPK has also been reported to be detrimental to heart function. With respect to cardiac hypertrophy, slight inhibition of p38 MAPK has been shown to promote myocyte growth via calcineurin-NFAT signaling. This study showed that the degree of calcineurin inhibition correlated with that of the p38 MAPK activity in the progressive growth response and myopathy in the heart [225]. An interesting study evaluating the difference between male and female mice in pressure overload-induced cardiac hypertrophy found that inhibition of the p38α MAPK augmented hypertrophic response to a greater extent in females [226].

MAPK has been observed to be intimately involved in cardioprotection pathways. In particular, high levels of p38 MAPK activity have been linked with cardioprotective effects during sustained ischemia [227]. Increases in p38 MAPK phosphorylation have also been shown to correlate with nicorandil recapturing of protection obtained from preconditioning *in vivo* [228]. When investigating the effects of different inhibitors of MAPK, it is important to consider the actions of solvent, as a water soluble SB203580.HCl did not affect ischemic-preconditioning or produce any protective action during ischemia, unlike previous studies which have not considered the anti-oxidant properties of DMSO, a common SB203580 solvent [229]. Nonetheless, other inhibitors of MAPK have revealed cardioprotective action. For example, SB202190, when co-administered with adenosine, has been shown to attenuate glycolysis and H^+^ production, increase glycogen synthesis, and improve the recovery of LV work [230]. Inhibition of MAPK using SB202190 has been reported to prevent increased Ca^2+^ uptake in cardiomyocytes and reduced Ca^2+^ release in SR caused by hypoxic preconditioning [231]. Another p38 MAPK inhibitor, FR167653, has also been observed to attenuate protection against I/R injury via nitric oxide triggered ERK1/2 and p38 MAPK activation, which is involved in cardioprotection [232]. Thus p38 MAPK has been suggested to be either a cardioprotective or detrimental kinase depending on the type of cardiac stress [215]. Activation of ERK, along with inhibition of p38 MAPK by FGF-2, has shown to be cardioprotective during I/R injury [233]. Selective p38 MAPK inhibitor, SC409, was found to produce cardioprotective effects by increasing left ventricular ejection fraction and cardiac output in mice with HF post MI [234]. Timing of treatment has also been shown to be crucial for the induction of cardioprotection, as demonstrated by a study investigating lethal ischemia. Mocanu *et al*. [227] found that p38 MAPK inhibitor, SB203580, is cardioprotective during lethal ischemia, but not during pre-conditioning. However, p38 MAPK phosphorylation has also been shown to correlate with preconditioning protection by reducing fragility in ischemically preconditioned myocytes [235]. This observation suggests that the timing of induced cardioprotection is important and that one should consider allowing p38 MAPK to become active to induce preconditioning in preventing contractile dysfunction post ischemic-reperfusion injury. 

## 7. Conclusions

From the foregoing discussion, it is evident that various protein kinases are involved in different signal transduction pathways in cardiomyocytes. These enzymes affect cardiac function by phosphorylating different sites engaged in the regulation of cation transport, cellular metabolism, cardiac growth and apoptosis, as well as contractile activity (Table 1) [236,237,238,239].

**Table 1:** Protein kinases and their substrates.

Protein Kinase	Substrates	References
Protein kinase A	PLB, RyR2, cTn1, cMyBP, L-type Ca^2+^ channels	[10,11,12,13,14]
Ca^2+^-calmodulin-dependent protein kinase	PLB, RyRs, L-type Ca^2+^ channels	[75,76,77]
Protein kinase C	AP-1, TGF-β, TnI, β-AR, adenylyl cyclase, GRK, L-type Ca^2+^ channel, RACKS, c-Raf	[236,237]
Phosphoinositide-3 kinase	Phosphatidylinositol, phosphatidylinositol (4,5)-bisphosphate	[238]
P38 mitogen-activatedprotein kinase	MKs, TFs, phospholipase A2, Tau, keratin 8, NHE-1	[239]

Abbreviations: PLB—phospholamban, RyR—ryanodine receptor, cTn1—cardiac troponin I protein, cMyBP—myosin binding protein, AP-1—activator protein-1, TGF-β—transforming growth factor-β, TnI—troponin I, β-AR—β-adrenergic receptor, GRK—G-protein coupled receptor kinases, RACKS—Receptor for activated C kinase, MK—mitogen kinase, TF—transcription factor.

A wide variety of pharmacologic agents have been used as experimental tools for inhibiting various protein kinases (Table 2) suggesting that these signal transducing proteins are viable targets for drug development for improving cardiac function in heart disease. 

**Table 2:** Protein kinases and their inhibitors.

Protein Kinase (Isoform)	Inhibitor	References
Protein kinase A	H89	[58,41,68,69]
	KT5720	[42,43,44]
Ca^2+^-calmodulin-dependent protein kinase	KN93	[96,97,100,101]
Protein kinase C	Breviscapine	[120]
	Chelerythine	[136]
	Gö6983	[131,132]
Protein kinase C(α)	Ro-320432	[106]
Protein kinase C(α)	Ro-318110	[106]
Protein kinase C(β)	Ruboxistaurin	[126,128,129]
PI3K	Wortmannin	[169,170,171,181]
P38 MAPK	SB203580	[197,201,203,206,207,215,216,220,222,228,230]
	SB202190	[209,231,232]
	FR167653	[214,216,233]
	SC409	[235]

CaMKII inhibition is effective in suppressing arrhythmias, as well as reducing hypertrophic response upon inhibition of the CaMKII δC isoform indicating that inhibition of CaMKII, particularly the δC isoform, would reduce cardiac dysfunction [86,92,93]. However, when considering inhibiting CaMKII, it may be important to inhibit calcineurin because of its involvement in cardiac dysfunction [102]. Activation of PKC in diabetic cardiomyopathy and cardiac hypertrophy reveals that different PKC inhibitors may prevent these diseases as some of these agents have been found effective in attenuating abnormal cardiomyocyte function [118,127]. However, inhibition of the PKC-ε isoform would also prevent its cardioprotective effects whereas inhibition of the PKC-δ isoform may augment cardioprotection [143,144,145]. Although delaying the development of cardiac dysfunction in HF and prevention of cardiac hypertrophy have been demonstrated upon inhibiting PI3K [166,167], it is emphasized that this signal transduction pathway is mainly involved in cardioprotection and thus its enhancement by various interventions has the potential for therapeutic use [178,183,184,185,186,187]. Finally, p38 MAPK inhibition has been shown to prevent cardiac remodeling, increase resistance to I/R injury, and improve myocardial function in HF [151,195,200,217,240,241]. With respect to cardioprotection, an increase in the activity of ERK1/2 MAPK was observed to correlate with increased level of cardioprotection [227], whereas its inhibition was found to attenuate its beneficial effects [231,232]. Recently, activation of ERK 1/2 was observed to be associated with cardiac hypertrophy and HF due to volume overload and these changes were attenuated by the blockade of the renin-angiotensin system [242]. Different protein kinases such as PKA, CAMKII and p38 MAPK have been suggested to regulate myocardial metabolism upon phosphorylating the mitochondrial site for palmitoyltransferase-1 activity [243]. Although tyrosine kinases were not discussed in depth in this review, these have been found to be effective in cardioprotection [244,245]. Tyrosine kinase-mediated phosphorylation has also been noted to attenuate apoptosis during cardioprotection which was prevented by its non-specific inhibitor, genistein [246]. In conclusion, although inhibitors of protein kinases may prove to be potential therapeutic agents in preventing and/or treating cardiac dysfunction, it is important to emphasize that inhibition of different protein kinase targets may prevent their cardioprotective effects.
